# Differential Effects of Tissue Culture Coating Substrates on Prostate Cancer Cell Adherence, Morphology and Behavior

**DOI:** 10.1371/journal.pone.0112122

**Published:** 2014-11-06

**Authors:** Michelle S. Liberio, Martin C. Sadowski, Carolina Soekmadji, Rohan A. Davis, Colleen C. Nelson

**Affiliations:** 1 Eskitis Institute for Drug Discovery, Griffith University, Nathan, Australia; 2 Australian Prostate Cancer Research Centre - Queensland, Institute of Health and Biomedical Innovation, Queensland University of Technology, Princess Alexandra Hospital, Translational Research Institute, Brisbane, Australia; Thomas Jefferson University, United States of America

## Abstract

Weak cell-surface adhesion of cell lines to tissue culture surfaces is a common problem and presents technical limitations to the design of experiments. To overcome this problem, various surface coating protocols have been developed. However, a comparative and precise real-time measurement of their impact on cell behavior has not been conducted. The prostate cancer cell line LNCaP, derived from a patient lymph node metastasis, is a commonly used model system in prostate cancer research. However, the cells’ characteristically weak attachment to the surface of tissue culture vessels and cover slips has impeded their manipulation and analysis and use in high throughput screening. To improve the adherence of LNCaP cells to the culture surface, we compared different coating reagents (poly-l-lysine, poly-l-ornithine, collagen type IV, fibronectin, and laminin) and culturing conditions and analyzed their impact on cell proliferation, adhesion, morphology, mobility and gene expression using real-time technologies. The results showed that fibronectin, poly-l-lysine and poly-l-ornithine improved LNCaP cells adherence and provoked cell morphology alterations, such as increase of nuclear and cellular area. These coating reagents also induced a higher expression of F-actin and reduced cell mobility. In contrast, laminin and collagen type IV did not improve adherence but promoted cell aggregation and affected cell morphology. Cells cultured in the presence of laminin displayed higher mobility than control cells. All the coating conditions significantly affected cell viability; however, they did not affect the expression of androgen receptor-regulated genes. Our comparative findings provide important insight for the selection of the ideal coating reagent and culture conditions for the cancer cell lines with respect to their effect on proliferation rate, attachment, morphology, migration, transcriptional response and cellular cytoskeleton arrangement.

## Introduction

In multicellular organism tissues the extracellular space surrounding cells is filled with a complex mixture of macromolecules referred to as the extracellular matrix (ECM). The ECM is composed of polysaccharides and proteins, such as laminin, fibronectin, elastin, collagen, and their relative amount is tissue specific. These proteins are embedded in a polysaccharide gel. [1] Despite the initial thoughts of serving merely as a scaffold for cells, it is now known that the ECM is not just structural but instructive, being responsible for regulating cellular behavior and affecting their proliferation, shape, function, migration, survival and development [2]–[5].

Many of the ECM proteins have important adherence function. [1] Most cells are anchorage-dependent and need to attach to the ECM in order to survive and proliferate. [6] Integrins are transmembrane proteins in the form of αβ heterodimers integral for the ECM protein-cell attachment. This interaction generates a cascade of intracellular signals that can also control differential gene expression. [7], [8] The signaling response is related to the ECM molecular composition that changes according to the cell response to their micro-environment. [9], [10] In this way, the ECM is in constant change to facilitate cell requirements of developmental plasticity. [11] Nevertheless, little is known about the molecular details involved in the signal transduction. The cell response to the ECM components is variable and dependent on which integrin subunits are expressed by the cells. Many research groups have been using different ECM proteins in tissue culture to modify cell behavior, primarily cell attachment. [12]–[15] However, in addition to increasing attachment, the coating proteins can affect other aspects of cell biology, influencing the final results of the assay [16].

The androgen-sensitive human prostate adenocarcinoma cell line, LNCaP, is one of the most commonly used model systems in prostate cancer (PCa) research. It was derived from a metastatic lesion in the lymph node of a 50-year old Caucasian male in 1977. [17] Weak cell-surface adhesion of cell lines is a common problem of tissue culture research and presents technical limitations to the design of experiments. Their characteristically weak attachment to the surface of tissue culture vessels and cover slips have impeded their manipulation, analysis and use in high throughput screening since LNCaP cells can be easily dislodged through modest mechanical forces like fluid shear stress. To improve the adherence of LNCaP cells to the culture surface, we compared different coating reagents (poly-l-lysine, poly-l-ornithine, collagen IV, fibronectin, and laminin) and culturing conditions, e.g. cell density, and analyzed their impact on cell proliferation, adhesion, mobility and morphology with a real-time cell analyzer (RTCA). Our findings are a helpful tool for the selection of the ideal coating reagent and culture conditions for the LNCaP cell line with respect to their effect on proliferation rate, attachment, morphology and cellular cytoskeleton arrangement.

## Materials and Methods

### Cell culture

LNCaP cells (American Tissue Culture Collection, Rockville, MD) were routinely cultured in RPMI growth media without phenol red (Invitrogen) supplemented with 10% (v/v) FBS (Invitrogen). LNCaP cells were propagated for no more than 40 passages.

### Coating conditions

All coating reagents were prepared as recommended by the manufacturers. The volume and concentration of the substances used for coating the wells were 1.3 µL laminin (LAM, 0.5 mg/mL in H_2_O, Invitrogen), 1 µL collagen from human placenta type IV (COL, 1 mg/mL in H_2_O, Invitrogen), 0.4 µL fibronectin (FN, 1 mg/mL in H_2_O, Invitrogen), and 0.32 µL poly-l-lysine (PLL, 1 mg/mL in H_2_O, Invitrogen). These coating reagents were mixed with H_2_O to a total volume of 50 µL per well of a 96-well plate. 50 µL poly-l-ornithine (PLO, 0.01% in H_2_O, Sigma-Aldrich) were directly added to the wells, and the plates were incubated overnight at 37°C in 5% CO_2_. The incubation time for LAM and FN was 4 h using the same conditions described above. The coated wells were washed once with DPBS followed immediately by cell seeding. The volume of coating substances was adjusted according to the growth area when different culture vessels were used.

### Real time cell analyzer (xCELLigence System)

The real time cell analyzer (RTCA) xCELLigence system (Roche Applied Science) comprises four main parts: the RTCA analyzer, the RTCA SP station, which stays inside a tissue-culture incubator, the RTCA computer with integrated software, and a 96-well E-plate. The bottom of the disposable 96-well E-plate is approximately 80% covered with gold microelectrodes that monitor the electronic impedance, detecting physiological changes of the cells. Cells in contact with the electrode will act as insulators, leading to an increase in impedance. Thus, the electrode impedance changes proportionally with alterations to number, size and adherence of cells growing in a monolayer [18], [19].

Changes in impedance are translated as the unitless term *cell index* (CI). CI = (Zi−Z0)/15, where Zi is the impedance at an individual point of time during the experiment and Z0 is the impedance at the start of the experiment. Thus, the CI is a quantitative and composite measure of the overall state of the cells in an electrode-containing well [18], [19].

First, 100 µL of complete medium were added to each well for measurement of the background. Then, LNCaP cells were seeded in a 96-well E-plate uncoated or coated with the indicated reagents as described above at a density between 9.4×10^3^ and 6.25×10^4^ cells/cm^2^ in triplicate. The E-plate was allowed to incubate at room temperature for 30 min and placed on the reader in the incubator for continuous recording of the cell index. The E-plate was incubated for 96 h at 37°C in 5% CO_2_, and the attachment of the cells was monitored via the CI for 4 h every 2 min. After this period, the CI was measured every hour for 92 h.

### Cell proliferation and viability

Cells were seeded in 96-well plates uncoated or coated with the indicated reagents at a density of 1.25×10^4^ cells/cm^2^. Growth as a function of increasing confluence was measured using the live content cell imaging IncuCyte HD system (Essen BioScience). Images were taken with a 10x objective at 2 h intervals from 3 separate wells per coating condition, and mean ± SD of confluence percentages was computed. Metabolic activity of the cells grown on the different coatings was measured with AlamarBlue after 96 h according to the manufacturer’s instruction (Invitrogen, USA). Kinetic analysis was performed with GraphPad Prism (GraphPad Software). Average values of triplicates were calculated after background correction.

### Adhesion and quantification of morphological parameters

LNCaP cells were seeded in a 96-well plate, uncoated or coated with the indicated reagents at a density of 3.12×10^4^ cells/cm^2^. After 24 h, 48 h, 72 h and 96 h the cells were fixed with 4% paraformaldehyde for 20 min on ice, permeabilized with 0.2% (v/v) Triton X-100/PBS for 10 min, and stained with CellMask Deep Red Plasma membrane Stain (2.5 µg/mL, Invitrogen) and 1 µg/mL DAPI (Invitrogen). The assessment of cell adhesion was performed measuring the number of cells left attached to the plate after the washing steps using the Operetta High Content Imaging System (PerkinElmer). The morphological parameters cell area and nuclei area were quantified.

### Time-lapse imaging

Surfaces of a 6 well/plate were coated as described above. LNCaP cells were seeded at a density of 1.58×10^4^ cells/cm^2^ and monitored for 96 h. Every 15 min an image was taken with a Zeiss Axio Observer light microscope (objective 20×) to follow shape changes and migration during time. The videos can be found as **Video S1–S6**.

### Distribution of F-actin

LNCaP cells were grown on glass cover slips uncoated and coated with the indicated reagents for 24 h and 96 h. Cells were then fixed and permeabilized as described above, followed by staining with rhodamine-phalloidin (1∶40, Invitrogen) and 1 µg/mL DAPI (Invitrogen). The immunofluorescence complexes were visualized with an Olympus (FV1000 Spectral) confocal microscope using a 60× lens. Optical sectioning was carried out by acquiring a stack of images at different focal positions along the z-axis.

### Scratch wound assay

LNCaP cells (3.12×10^4^ cells/cm^2^) were seeded in a 96-well Essen ImageLock plate (Essen BioScience) uncoated or coated as described previously, and were grown to confluence in a CO_2_ humidified incubator. After 24 h, the scratch was made using the 96-pin WoundMaker (Essen BioScience). Wound images were taken every 1 h for 36 h, and the data were analyzed by the integrated metric Relative Wound Density part of the live content cell imaging system IncuCyte HD (Essen BioScience). The experiment was done in triplicate.

### Sensitivity to simvastatin

The influence of FN, PLO and PLL coating on the cell sensitivity to simvastatin was investigated. LNCaP cells were seeded in triplicate and grown in a 96-well E-plates as described above. After 24 h incubation, the cells were treated with different concentrations of simvastatin (Sigma-Aldrich) and monitored every 1 h for 60 h using the RTCA system. The IC_50_ for 24 h, 48 h and 72 h treatment were calculated using the software GraphPad Prism 5 (GraphPad Software).

### qRT-PCR

Surfaces of a 6 well-plate were coated as described above, and LNCaP cells were seeded at a density of 1.05×10^4^ cells/cm^2^. After 72 h, the growth media was substituted by charcoal-stripped (androgen-depleted) serum RPMI media (CSS, Invitrogen, USA) supplemented with 5% (v/v) CSS and the cells were cultured for 48 h. Finally, LNCaP cells were treated with 20% (v/v) ethanol as control or the androgens R1881 (1 nM) and DHT (10 nM) for 30 h.

Total RNA was obtained using the RNeasy mini kit (Qiagen, USA) according to the manufacturer’s instructions. The quantity and the quality of the RNA were measured using a NanoDrop UV spectrophotometer (ThermoFisher Scientific, USA). Samples with a 260/280 ratio higher than 2.0 were used for subsequent procedures. The samples were treated with DNAse Amp grade I, and 2 µg of total RNA was reverse-transcribed using the cDNA synthesis method for the qPCR kit (Invitrogen). QRT-PCR was performed with SYBR Green master mix (Invitrogen) using the 7900HT Fast Real-Time PCR System (Applied Biosystems). Data were analyzed with SDS2.3 software (Applied Biosystems). The mRNA expression levels were calculated by the ΔΔCt method and normalized relative to the expression levels of the house keeping gene (GAPDH or RPL32) of the respective treatment and calculated relative to the ethanol uncoated control. The sequences of the primers used are listed in **Table S1**.

## Results

### FN, PLO and PLL improve cell-substrate adherence

**Figure 1:**
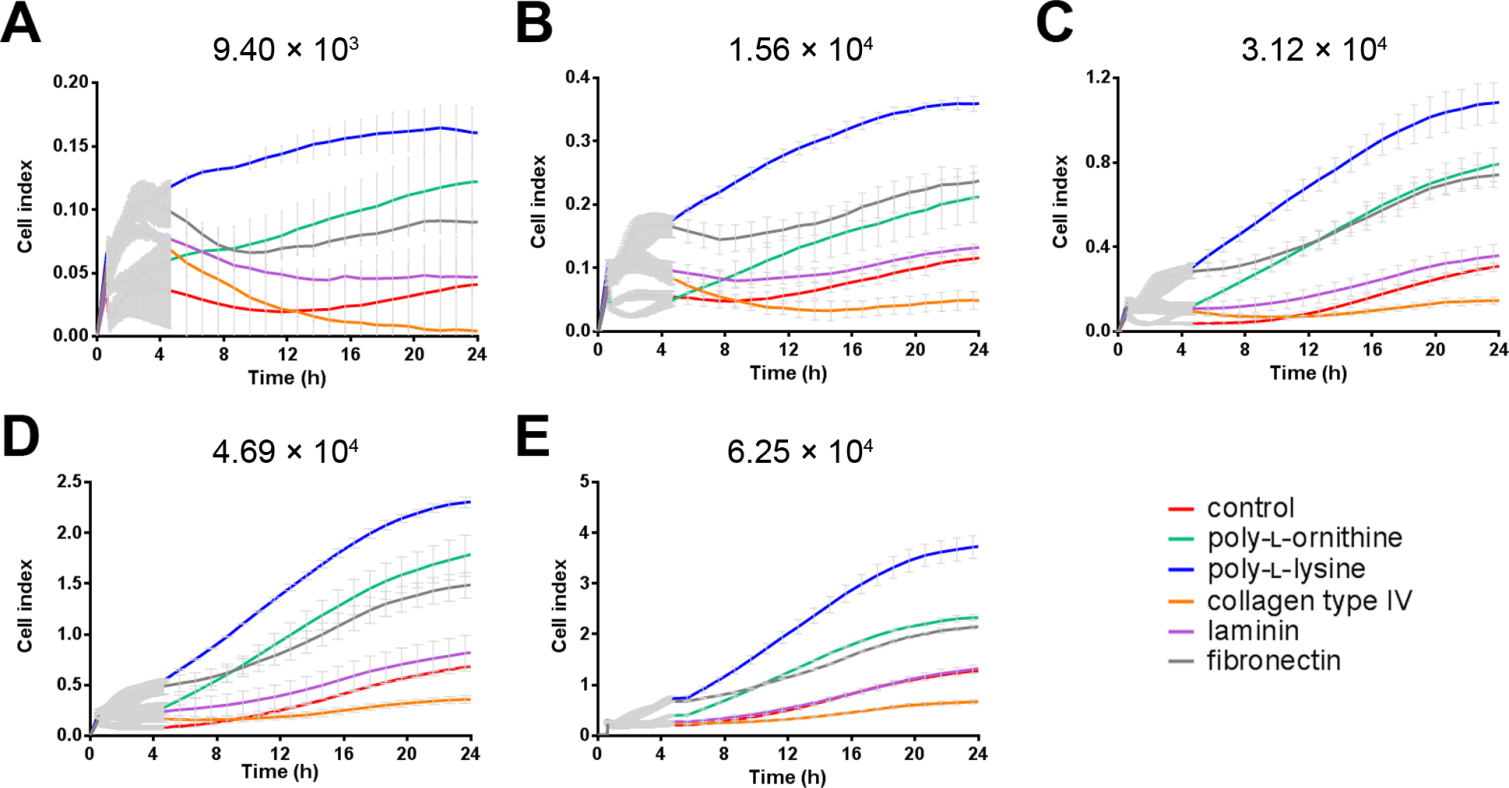
Coating and cell density effects on cell-substrate adherence of LNCaP cells. The cells were monitored for 24 h to analyze cell-substrate adherence from the time cells were added to the wells (t = 0) using a real-time cell analyzer (xCELLigence, Roche). Each data point is represented by its means (n = 3) ± SD.

**Figure 2:**
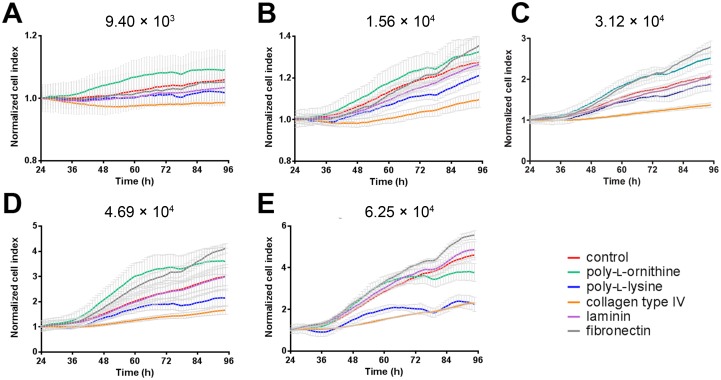
Coating and cell density effects on proliferation rate and morphology of LNCaP cells. The cells were monitored for 72 h to analyze the effects of the coatings on LNCaP cells from 24–96 h using a real-time cell analyzer (xCELLigence, Roche). The data was normalized at 24 h for comparison of the slopes. Each data point is represented by its means (n = 3) ± SD.

The real time cell analyzer (RTCA) xCELLigence is a label-free methodology that measures proliferation rate, adherence and morphology based on impedance changes. Changes in impedance are translated as the unitless term *cell index* (CI). We performed RTCA analysis of LNCaP cells seeded in wells pre-treated with the different coatings at cell densities between 9.4×10^3^–6.25×10^4^ cells/cm^2^ in a 96 well plate, and investigated both the attachment phase (24 h post seeding) (**Fig. 1**) and the proliferation phase (24 h to 96 h post seeding) of the cell culture (**Fig. 2**). It was assumed that the attachment of cells out of suspension onto the substrate and the cell morphology changes associated with this process were the major contributors to the CI during the first 24 h of the experiment (**Fig. 1**). Hence, the contribution of proliferation to the CI for this period was considered marginal, which was further supported by the fact that LNCaP cells grown under similar conditions displayed a doubling time of 36 h. [20] Indeed, comparison of the different seeding densities of the control and the coating reagents at 3.12×10^4^ cells/cm^2^ in a 96 well plate after 24 h revealed that PLL increased the CI to a similar extent as doubling the number of seeded cells, i.e. from 3.12×10^4^ to 6.25×10^4^ cells/cm^2^ (**Fig. S1**). At all cell densities, coating with fibronectin (FN) resulted in the highest CI after 24 h, followed by poly-l-lysine (PLL) and poly-l-ornithine (PLO). PLL and PLO increased the CI at very similar rates up to 3.12×10^4^ cells/cm^2^, while PLO decreased the slope at all cell densities (**Fig. 2**). The laminin (LAM) coating did not affect the CI when compared to control, while collagen type IV (COL) caused the CI to rise slower. Interestingly, this order was not affected by increasing the number of seeded cells. These findings suggested that FN, PLL and PLO markedly improved the attachment of LNCaP cells, with the ECM protein being superior to the poly-amino acids.

The proliferation phase was monitored from 24 h after seeding the cells to 96 h (**Fig. 2**). The major contributors of this phase to changes of the CI are cell proliferation, adherence and morphology. At all cell densities tested, LNCaP cells grown on LAM displayed a rate of CI increase that was indistinguishable from that of control. In contrast, LNCaP cells grown on COL substrate showed a lag phase where the CI did not increase up to 48 h after seeding, and an overall reduced rate of CI rise (**Figs. 2B–E**). These effects were independent of the number of seeded cells. At all cell densities the PLL substrate caused a detectable slowdown in the CI increase. The same effect was visible on PLO substrate at the highest seeding density (6.25×10^4^ cells/cm^2^, **Fig. 2E**), while the CI increased faster on PLO-coated substrate at lower seeding densities (**Fig. 2A and B**). Apart from the lowest cell density (9.4×10^3^ cells/cm^2^), the CI rate increase of cells grown on FN substrate were consistently higher than control (**Figs. 2B–E**); an effect which appeared to be unaffected by increases in the number of seeded cells. Taken together, high cell densities (>4.69×10^4^/cm^2^) negatively affected the CI when LNCaP cells were grown on substrates coated with poly-amino acids (PLL and PLO) but were unaffected with ECM proteins (COL, LAM and FN). This observation is of particular importance for cell culture experiments where a high cell confluence is desirable. Furthermore, a seeding density of 9.4×10^3^ cells/cm^2^ was overall detrimental to cell culture of LNCaP cells, resulting in lack of cell proliferation, which was probably due to a scarcity of cell-cell contacts.

### All coating conditions reduced cell proliferation but did not strongly affect LNCaP cell viability

**Figure 3:**
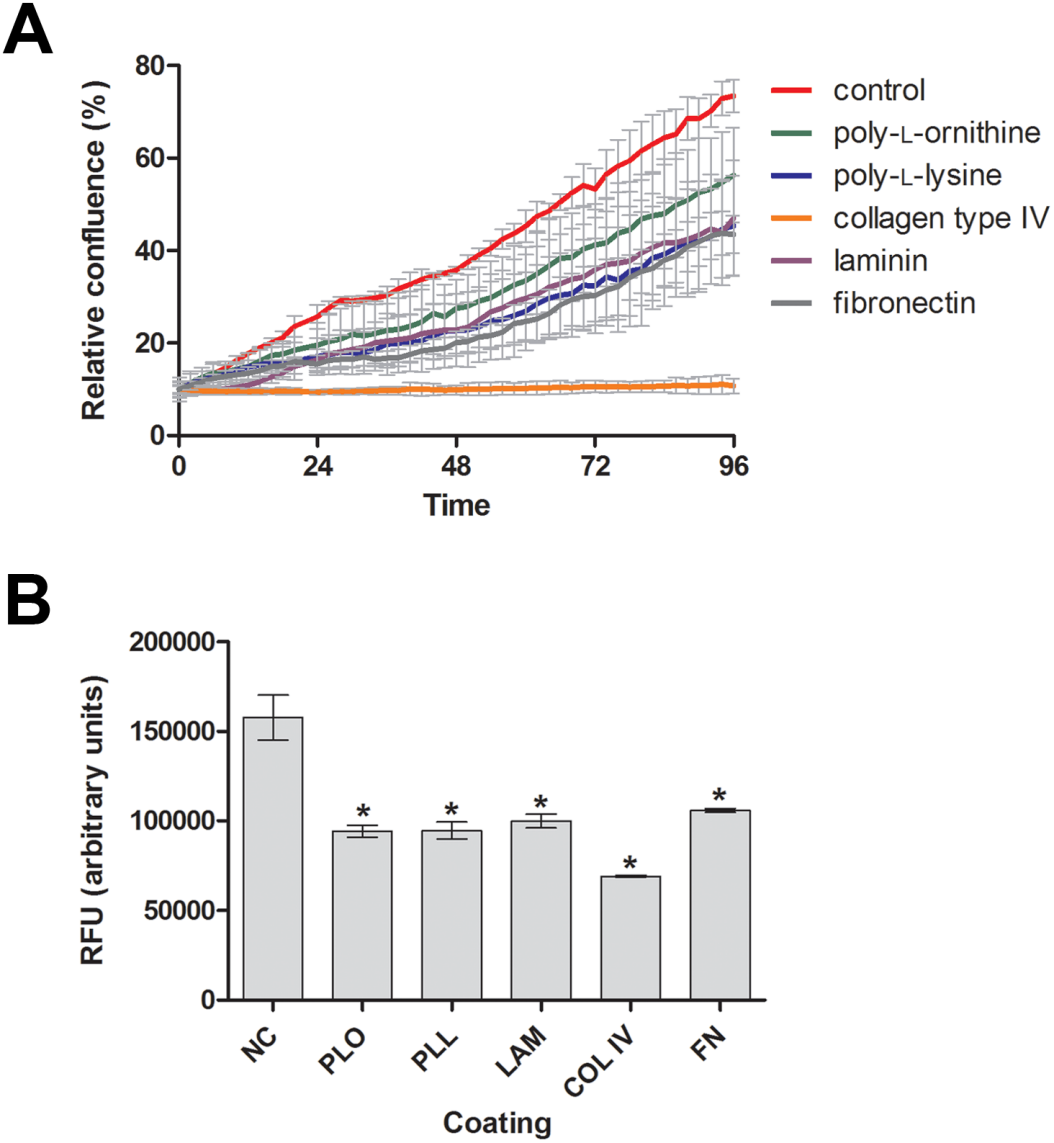
Relative confluence and cell viability of LNCaP cells seeded on wells pre-coated with different substances over a period of 96 h. LNCaP cells were seeded at 1.25×10^4^ cells/cm^2^ on a polystyrene 96 well-plate uncoated or coated with poly-l-ornithine, poly-l-lysine, collagen type IV, laminin or fibronectin. (**A**) Wells confluence was monitored every 2 h for 96 h using IncuCyte system. (**B**) Metabolic activity/cell viability was measured by AlamarBlue assay after 96 h. Each data point is represented by its means (n = 3) ± SD. Significant results (p<0.05) are marked with an asterisk.

The cell index is a combined measure of the proliferation rate, adherence and morphology of the cells. Hence, the effects of the coating reagents on each of these parameters were investigated separately. The cell density of the coated wells increased slower than the uncoated wells. Cells grown on FN, PLL, PLO and LAM displayed similar growth rates (**Fig. 3A**). Collagen type IV was the coating substance that negatively impacted cell proliferation the most. Examination of viability/metabolic activity of LNCaP cells grown for 96 h on the different coating substrates by AlamarBlue assay revealed that all coating reagents reduced cell viability, with COL slightly worse than the other coatings (**Fig. 3B**). This effect was similar to the results obtained for well confluence on different coatings (**Fig. 3A**). Taken together, these results showed that the coating reagents affected cell proliferation and metabolism activity of LNCaP cells.

### COL and LAM induced LNCaP cells to aggregate

**Figure 4:**
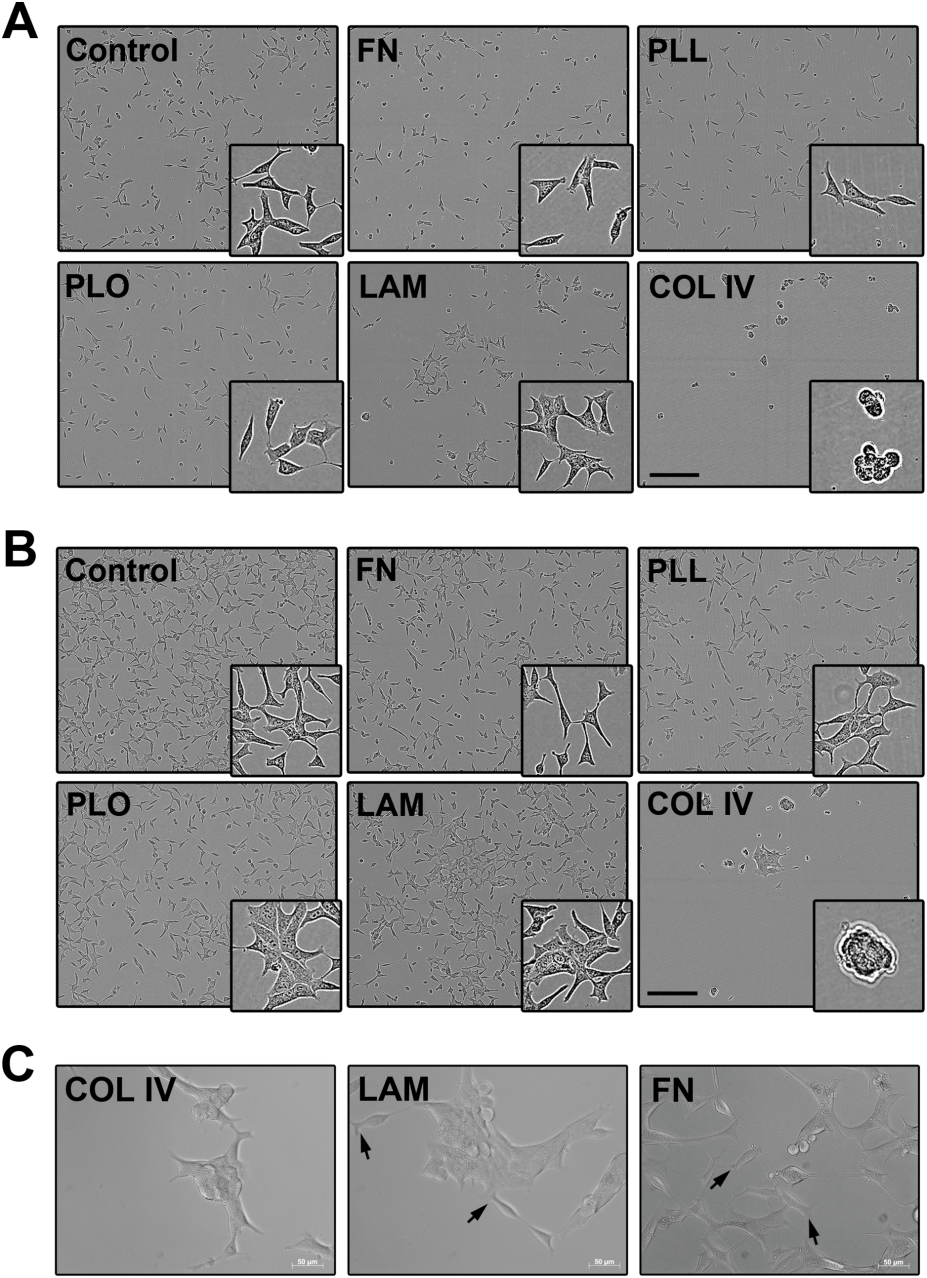
Morphology of LNCaP cells grown on different coated polystyrene well substrates. LNCaP cells imaged after 24 h (**A**) and 96 h (**B**) with IncuCyte system, 10×. Scale bar = 300 µm. (**C**) Snap shots from the 96 h time-lapse microscopy video of LNCaP cells. Arrows indicate lamellipodia of polarized cells. Scale bar = 50 µm (20×, Zeiss Axio Observer).

IncuCyte images revealed that LNCaP cells were attached to the surface in all coating conditions after 24 h (**Fig. 4A**). The cells grown in wells pre-coated with FN, PLL, PLO, LAM, as well as the control displayed a fibroblast-like shape typical for LNCaP cells. [21] In contrast, LNCaP cells cultured on COL had a rounder shape. Cells grown on COL and LAM also tended to aggregate. In addition, the wells coated with LAM and FN contained more round cells when compared to the other coating substrates (**Fig. 4A**). The same was observed after 96 h (**Fig. 4B**). After 96 h, most of the cells acquired a spindle shape in all coating conditions. Cells on COL grew in aggregates at 96 h (**Fig. 4B**).

In order to investigate the effect of the coating reagents on cell mobility and morphology more detailed in real-time, LNCaP cells were studied by high magnification time-lapse microscopy. Similar to the IncuCyte experiment, time-lapse microscopy showed that COL and LAM (**Fig. 4C**) caused LNCaP cells to form aggregates. In addition, cells grown in LAM-coated wells displayed the presence of numerous polarized cells, characterized by the presence of asymmetric cells. The PLL- and PLO-treated samples displayed a similar morphology when compared to the control. Yet, LNCaP seeded on these substrates seemed to attach faster than the control cells and migrate less. Videos of the time-lapse microscopy over a period of 96 h can be found in **Video S1–S6**.

### LNCaP cells attach better to surfaces coated with FN, PLO and PLL, which also affect cell morphology

**Figure 5:**
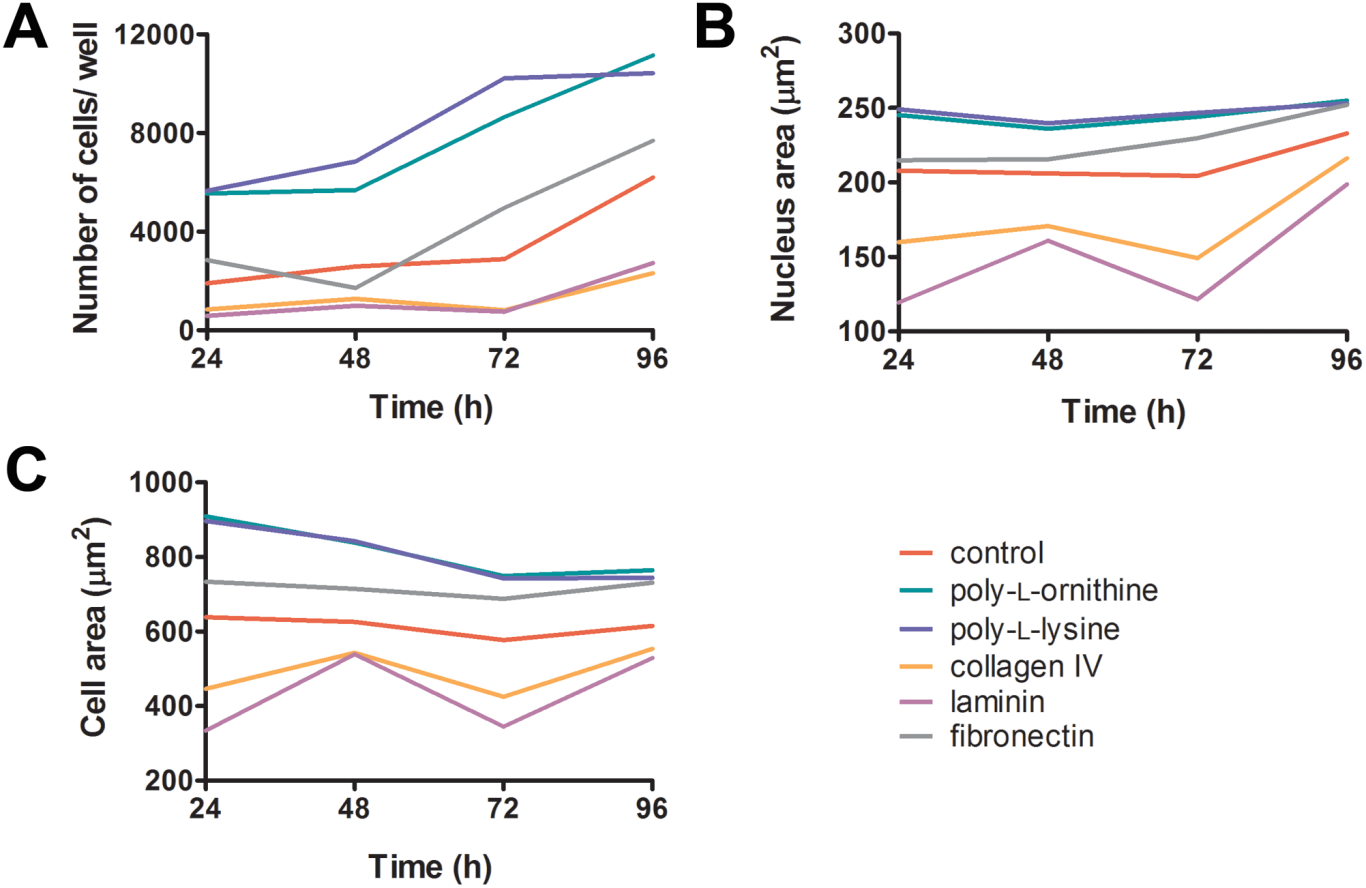
Quantification of morphological parameters and cell density of LNCaP cells (3.12×10^4^ cells/cm^2^) seeded on indicated coating reagents. Cells were analyzed for cell density (**A**), nuclear area (**B**) and cellular area (**C**) at four different time points using a high content screening instrument (Operetta).

Attachment and cell morphology, which compose part of the CI, were investigated by high content screening (HCS). HCS of LNCaP cells measured a substantially higher cell density (better attachment) after pre-treatment of wells with PLO or PLL when compared to control, FN, COL or LAM at all time points (**Fig. 5A**). Cells cultured in the presence of FN attached better than the control cells at 72 h and 96 h. In regards to cell morphology changes, the area of the nucleus and the total cell area were affected by all coatings (**Figs. 5B and 5C**). Cells cultured on PLO, PLL and FN for 96 h showed increased nuclear and cellular areas of at least 8% and 18%, respectively, when compared to control. Relative to the control, COL and LAM decreased the nuclear and cellular areas by 7% and 10%, and 15% and 14%, respectively.

### The different coating reagents affected F-Actin organization

**Figure 6:**
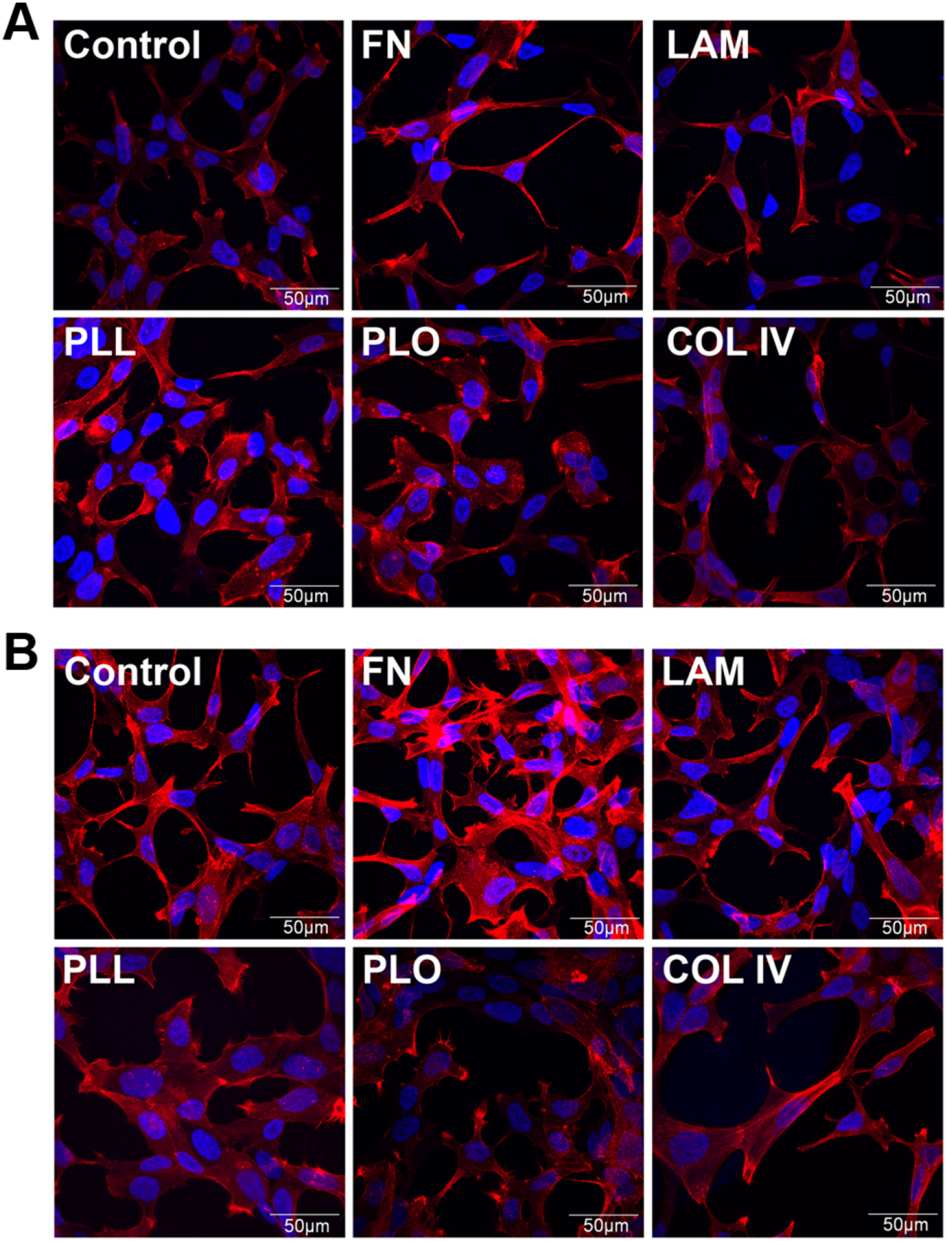
Immunofluorescence of LNCaP cells stained for F-actin. Cells were grown on glass cover slips without coating (control), or coated with fibronectin (FN), laminin (LAM), poly-l-lysine (PLL), poly-l-ornithine (PLO), or collagen type IV (COL IV). After 24 h (A) or 96h (B), cells were stained for F-actin with rhodamine-phalloidin, counterstained with DAPI, and analyzed by confocal fluorescence microscopy (60×, Olympus). Arrows indicate lamellipodia of polarized cells and arrow heads mark filopodia. Scale bar = 50 µm.

To investigate the changes in cell morphology and mobility of LNCaP cells in more detail, we performed confocal fluorescence microscopy and investigated the F-actin organization of LNCaP cells, such as the presence of lamellipodia at 24 h **(** **Fig. 6A** **)** and 96 h **(** **Fig. 6B** **)** post-seeding. These structures consist of parallel-bundled actin filaments that probe the substrate to decide where and how the focal adhesions should be established for attachment. In addition, these filaments contribute to the formation of actin stress fibers. [22], [23] The adhesion and spreading of cells involves the remodeling of the cytoskeleton. Dynamic structures called focal contacts form around integrins at the adhesion sites. The integrins are bound to ECM components on one side, and to actin filaments called stress fibers on the other side. The application of force in one side cause reaction on the other, and this, together with integrin signaling pathways, determines the cell shape. [3] Consistent with the results observed in the time-lapse microscopy experiment, we observed many polarized cells after 24 h on FN- and LAM-treated glass cover slips (**Fig. 6A**). In addition, after 96 h it was observed that the fibers became more disorganized with some cortical actin accumulation around the cell body and a reduced number of stress fibers in the presence of FN (**Fig. 6B**). Furthermore, FN caused a substantial increase in actin staining, and the cells lost their polarity (**Fig. 6B**).

Cells grown in the presence of PLL and PLO (**Fig. 6**) displayed a more diffuse actin pattern with some concentrated actin staining at the cell periphery at 24 h and 96 h. The presence of many actin bundles and radially extended actin filaments around the cells, which are called filopodia, were observed. The nuclei of the cells cultured on PLL displayed a strong DAPI intensity at 24 h (**Fig. 6A**), which was reduced at 96 h (**Fig. 6B**). Moreover, the nuclei increased in size after 96 h.

At 24 h, the cells in the wells coated with COL (**Fig. 6A**) showed an actin pattern similar to the control. Nevertheless, after 96 h of growth, the actin filaments became more organized than in the control (**Fig. 6B**).

### PLL, PLO, or FN-coated wells increase the attachment of LNCaP cells and slightly decreased cell mobility, while LAM increases migration

**Figure 7:**
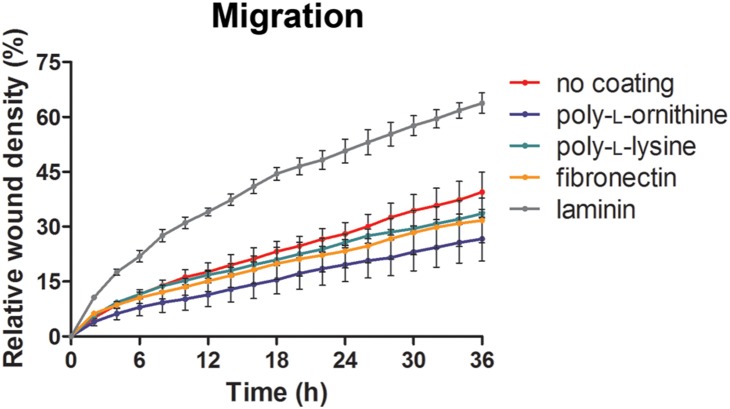
Wound healing assay by live cell imaging on the IncuCyte system. Relative wound density at different time points of LNCaP cells over a period of 36 h. The measurements are from wounds made on a monolayer of LNCaP cells cultured in the presence of different coating treatments and control.

Morphological changes are usually associated with alterations of other cell characteristics, including motility, differentiation and metabolic activity. [24] To address this possibility, the motility of LNCaP cells grown on polystyrene treated with the different coating reagents was assessed in a wound healing assay (**Fig. 7**). LNCaP cells were seeded for 24 h in wells coated with FN, LAM, PLL, PLO or COL, and the cell monolayer was scratched using a 96-pin WoundMaker. The quality of the wounds generated on PLL, PLO, or FN-coated wells were superior to the control (no coating) and LAM, as judged by the relative smoothness of the edges of the scratch as well as a very similar wound area (**Fig. S2**). Another observation was that LNCaP cells cultured in PLO or PLL-treated wells colonized the well in a semi-organized pattern, where the elongated cell bodies were aligned in parallel (**Fig. S2**). Pre-treatment with LAM did not improve the adherence of LNCaP cells when compared to the control, and the WoundMaker generated wounds with uneven edges and of different area. LNCaP cells dislodged as large sheets of cells from COL-treated wells when processed with the WoundMaker (data not shown), indicating that COL was inferior to uncoated wells and not suitable for this application. Analysis of the relative wound density showed that cells grown in the presence of LAM migrated 62% faster into the wound area than the control cells after 36 h (**Fig. 7**). In comparison, PLL, PLO and FN caused LNCaP cells to migrate slower than the control, displaying a reduction of wound density by 15%, 33% and 20% grown under these conditions respectively. Notably, LNCaP cells displayed a doubling time around 36 h, as observed in the RTCA experiments, [20] suggesting that the observed effects on the wound density were caused predominantly by cell migration and not proliferation.

### PLL sensitizes LNCaP cells for the HMGCR inhibitor simvastatin

Previous studies have shown that the pre-coating with ECM components can affect the sensitivity of cells to various drugs. For instance, LAM and FN have been reported to increase resistance to ionizing radiation and to the cytotoxic drug Ukrain in human tumor and normal cells *in* *vitro*. [25] Furthermore, it was reported that adherence to a FN substrate induced cholesterol synthesis through activation of HMGCR and also increased fatty acid synthesis in human fibroblasts and rat hepatoma cells, while a PLL substrate or FN in solution had no effect on these pathways [26].

Hence, the effect of the coating reagents PLL, PLO and FN on the sensitivity of LNCaP cells to the HMGCR inhibitor simvastatin was investigated by RTCA, and the IC_50_ was calculated for treatment periods of 24 h, 48 h, and 72 h (**Fig. S3**). LAM and COL effects were not analyzed because these coating reagents had adverse effects on LNCaP cell-surface adherence and caused cell aggregation. While the IC_50_ for simvastatin was relatively similar among the four coating conditions at 24 h, PLL increased the sensitivity of LNCaP cells to the HMGCR inhibitor by two-fold after 48 h of treatment when compared to control (**Fig. S3**). At 72 h, LNCaP cells grown on a PLL substrate displayed a three-fold lower IC_50_. Similarly, PLO and FN increased the sensitivity of LNCaP cells to simvastatin by two-fold relative to the control at 72 h. However, it has been observed that PLO, PLL and FN reduced the cell viability by one third at 96 h (**Fig. 3B**). Hence, the sensitization of LNCaP cells by PLO and FN to simvastatin might be actually the effect of these coatings on cell viability. In this case, only PLL may significantly sensitize LNCaP cells for the HMGCR inhibitor simvastatin in a time-dependent manner.

### Androgen responsiveness and AR signaling were in general not affected by the coating conditions

**Figure 8:**
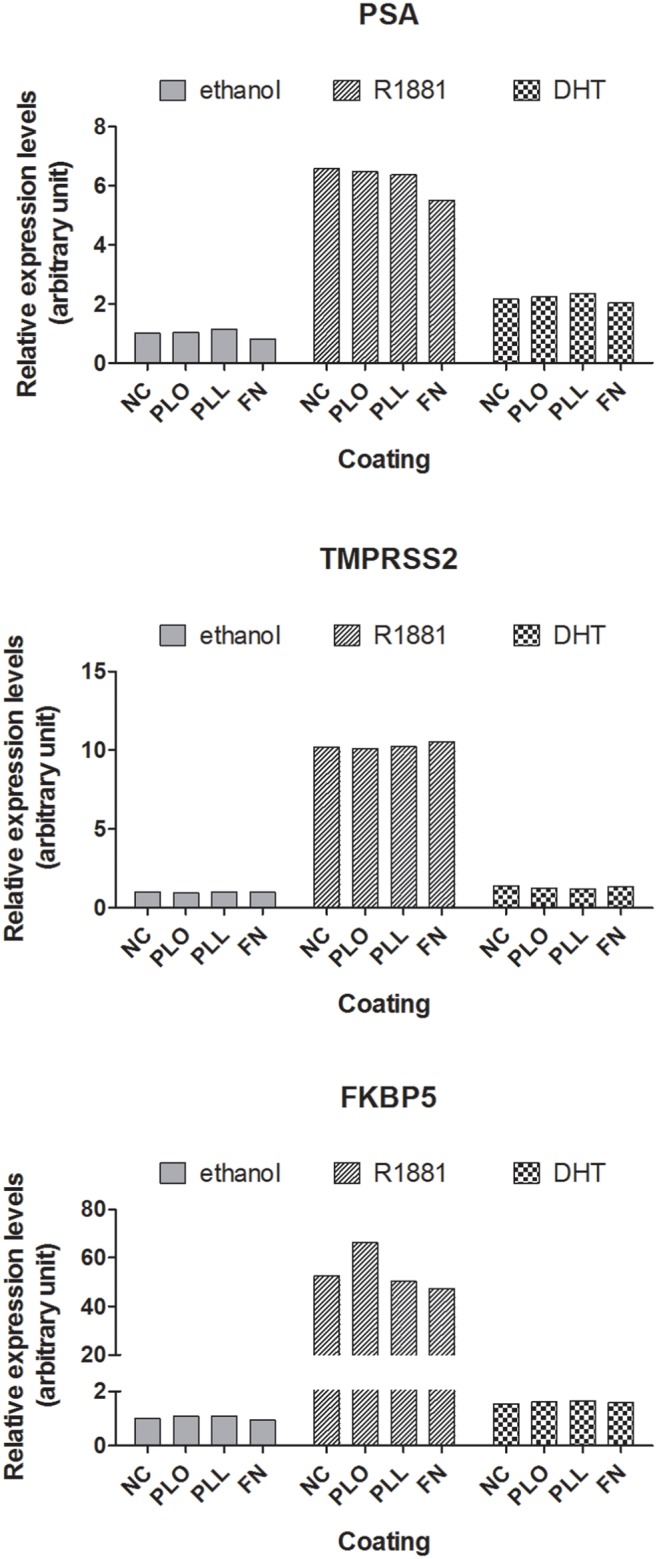
Relative expression levels of classic AR-regulated genes in response to androgen treatment in the presence of different coatings. LNCaP cells were grown on uncoated (NC) or coated wells (PLL, PLO or FN) in androgen-depleted medium for 72 h before androgen treatment with R1881 (1 nM) and DHT (10 nM) for 30 h. The expression levels of the indicated genes were analyzed by qRT-PCR, normalized to the housekeeping gene GAPDH and calculated relative to the ethanol uncoated control (NC).

In the healthy human adult prostate epithelium, AR expression and cell adhesion to the substratum occur in separate cell layers, namely in luminal and basal cells. Hence, the pathways of AR signaling and cell adhesion are unlikely to interact directly. [29] During the development of prostate cancer, malignant luminal epithelial cells change from cell-cell adhesion to cell-substratum adhesion, and signals from cell adhesion and AR are co-expressed. [27] The LNCaP cell line is an important model system to study androgen receptor (AR)-mediated signaling in prostate cancer. It was recently shown that changes to cell-cell contacts and the extracellular matrix altered the response of LNCaP cells to androgens. [28] Hence, it was important to investigate if the coating reagents FN, PLL and PLO, which increased LNCaP adherence, affected AR signaling in this model system. To address this issue, the expression of genes regulated by androgens was examined by qRT-PCR. [28] As shown in **Figure 8** for the classic androgen-regulated genes PSA [29], TMPRSS2 [30] and FKBP5 [28], androgen-depleted LNCaP cells displayed a typical response to androgen treatment with DHT or the synthetic androgen R1881 by an up-regulation of gene expression when compared to control. Furthermore, analysis of additional androgen-regulated genes derived from a gene set of 6598 genes commonly regulated by androgens (DHT and R1882) showed no significant differences in their differential expression (**Fig. S4**). [28] In summary, androgen responsiveness and AR signaling were in general not affected by growing LNCaP cells on PLO, PLL or FN substrates. **Table 1** summarizes the effects of the different coating substrates on androgen responsiveness and on other cellular parameters investigated in this study.

**Table 1:** Summary of the general effects of the coating substances on different cellular parameters.

Cellular parameter	Poly-l-lysine	Poly-l-ornithine	Fibronectin	Laminin	Collagen type IV
Abbreviation	PLL	PLO	FN	LAM	COL
Attachment	**↑**	**↑**	**↑**	**↓**	**↓**
Proliferation	**↓**	**↓**	**↓**	**↓**	**↓**
Cell viability	**↓**	**↓**	**↓**	**↓**	**↓**
Cellular area	**↑**	**↑**	**↑**	**↓**	**↓**
Nuclear area	**↑**	**↑**	**↑**	**↓**	**↓**
Migration	**↔**	**↓**	**↔**	**↑**	nd
Actin organization	**↓**	**↓**	**↓**	**↔**	**↑**
Actin staining	**↔**	**↔**	**↑**	**↔**	**↔**
Polarized cells	**↓**	**↓**	**↓**	**↑**	**↔**
Cell aggregation	**↔**	**↔**	**↔**	**↑**	**↑**
Androgen response	**↔**	**↔**	**↔**	nd	nd
Sensitivity to simvastatin	↑	**↔**	**↔**	nd	nd

The results below were obtained by the analysis of the last time point of different experiments. Nd = not determined; ↓ = decrease; ↑ = increase; **↔** = no change observed.

## Discussion

Most epithelial derived cells are anchorage-dependent. Their attachment to a surface is mandatory for viability and proliferation, and detachment induces cell death through the process of anoikis. Attachment is also necessary for events such as cell spreading, cell migration and differentiation. [13] The use of ECM components such as FN, COL and LAM to improve cell attachment is a common practice in tissue culture and may be essential for high throughput screening where liquid shear forces can disrupt cell attachment. The use of other substances such as poly-amino acids and different textures of the substrate surface are alternative strategies used to improve the adherence of cells. Nevertheless, an increase in cell-substrate adherence can affect various aspects of cellular behavior. For example, cell surface receptors (integrins) that mediate the cell attachment also control processes such as survival, proliferation, differentiation and migration [7].

The prostate cancer cell line LNCaP is a popular model system to study androgen-regulated pathways critical for this disease. Nevertheless, use of LNCaP cells in applications like siRNA-mediated gene silencing, immunofluorescence microscopy, wound healing, and high content screening, which involve tissue culture manipulations that generate mechanical forces like fluid shear stress is currently negatively impacted by their weak attachment to polystyrene and glass surfaces. To facilitate the use of LNCaP cells in these assays, different coating reagents and seeding densities were tested. Three ECM proteins (FN, COL and LAM) and two poly-amino acids (PLL and PLO) were used to pre-coat the wells before cells were seeded. The RTCA instrument measures the CI of the cell culture in real time based on changes to the impedance, which is influenced by adherence, cell morphology and cell number. Hence, in a proliferating cell culture the CI increase over time is mainly due to an increase in the cell number. Nevertheless, RTCA results need to be interpreted with caution because major changes to cell morphology and adherence can give a misleading understanding of the proliferative status of a cell culture. This was highlighted by the first 24 h of the RTCA experiment where coating-mediated changes in cell adherence increased the CI to the same extent as a doubling of the cell number. Hence, the RTCA experiment was divided into two phases, the attachment phase and the proliferation phase. Monitoring of the attachment process in real time revealed that FN, PLL and PLO markedly improved adherence of LNCaP cells. Notably, the ECM protein (FN) was superior to the relatively unspecific poly-amino acids (PLL and PLO). An accelerated increase of the CI in the presence of FN was previously reported for NIH3T3 and ND7/23 cells. [31], [32] The effect of all five coating reagents on LNCaP cells during the attachment phase was not impacted by alterations in the seeding cell density. However, this was not the case during the proliferation phase, i.e. substrate coated with PLO negatively affected the CI when LNCaP cells were seeded at high cell numbers (>3.12×10^4^ cells/cm^2^) and PLL decreased CI slope at all cell densities. PLL has been previously shown to slow down the rising CI over time in NIH3T3 cells. [31] This density-dependent effect was not observed with ECM proteins (COL, LAM and FN). This observation is of particular importance for cell culture experiments where a high cell confluence is desirable. Furthermore, the results shown here advise against a too low seeding density (9.4×10^3^ cells/cm^2^) and recommend a seeding cell number between 1.56×10^4^ and 3.12×10^4^ cells/cm^2^ in a 96-well plate. The measurement of cell density in a live content imaging experiment revealed that the CI increase observed in the presence of PLO and FN were not due to a positive effect on cell proliferation. Actually, the proliferation rate slightly decreased in the presence of coating. Hence, the phenomenon observed was a result of an increase in cell adherence and/or morphology changes. The decrease in CI observed with COL-coated polystyrene could be explained by the clustering of cells into aggregates, the round cell morphology and the reduction in the proliferation rate. This phenomenon was also observed with platelets cultured in the presence of collagen type IV. [33] HCS data confirmed the smaller cellular area and also the weak attachment of the cells to the COL substrate. LNCaP cells dislodged as a sheet of cells during the scratch making, and it was not possible to obtain a useful scratch on COL substrate. In contrast, the data obtained from confocal fluorescence microscopy indicated that the cells grown on COL-coated glass attached well to the substrate and displayed a morphology that was similar to the control. An increase in stress fibers and in lamellipodia for the cells cultured on COL was also observed, suggesting increased cell mobility. The structures observed are the result of integrin-mediated cell adhesion that re-organizes the actin cytoskeleton of the cells. This event comprises the recruitment of signaling complexes to the membrane. [7] The disparity between the results with COL coating could be due to the different surface substrate material used (polystyrene *versus* glass). It has been previously shown that different substratum characteristics, including surface charge, topography, hydrophobicity or hydrophilicity, surface chemistry and surface energy may influence cell behavior. [34] The characteristics of the substrate may also affect the polymerization/conformation of the ECM protein that could exhibit different binding sites to interact with integrins. Thus, the modified cell-substrate interaction could affect the generation of intracellular signals. [14], [35], [36] The experiments using RTCA, HCS and phase-contrast microscopy were performed with polystyrene plates, while glass cover slips were used for confocal fluorescence microscopy. Moreover, the response of cells to diverse textures is different than when they are on a smooth surface. [13] It has been demonstrated that rough surfaces are advantageous for cell attachment. This fact is continuously used in the development of osteoimplants [37]–[39].

LAM did not affect the CI compared to control. However, LAM decreased the cell proliferation rate and the adherence of LNCaP cells, as shown by the slightly worse scratches during the wound making process when compared to the control. Despite this, calculation of the relative wound density revealed that cells grown on LAM substrate migrated into the wound much faster than the control or any of the other coatings. This increased cell mobility was also observed by time lapse microscopy and was also indicated by the large number of polarized cells with expanded filopodia.

The effect of FN on the CI was mainly due to an improvement in adherence. LNCaP cells seeded on FN quickly attached to the substrate as observed by time-lapse microscopy. These findings were supported by the increase in cell area observed by HCS, and by the increase of cellular F-actin and filopodia. Despite the increase in stress fibers and lamellipodia, FN reduced the mobility of LNCaP cells, which may be related to the increase in attachment of the cells to the substrate and to the reduction in polarization. The observed decrease of LNCaP cell proliferation with FN is in accordance with literature data [40].

As shown by HCS, F-actin staining and wound assay, PLO and PLL improved adherence of LNCaP cells to polystyrene and glass when compared to control. It is the first time that PLO has been reported to increase the adherence of LNCaP cells. The use of this poly-amino acid has been preferable to PLL in some applications for being less immunogenic. [41] The flat morphology of the cells and the high intensity of F-actin staining could be observed on the confocal images. Furthermore, it is noteworthy to mention the presence of many filopodia around the cells. Work by the Faix lab demonstrated that the number of filopodia is directly proportional to the dDia2 protein level in the cell. [42] Abundant filopodia have been linked to invasive phenotype in cancer cells when most of the filopodia are found at the lamellipodia of a migrating cell but not all around the cell like in our data. [43] The finding that PLL and PLO improved adherence was further supported by a reduction in cell migration as seen in the wound healing assay and time lapse microscopy. A correlation between strong cell adhesion and reduced mobility has been noted when NHK cells are grown on laminin-332 matrix. [44] The cells also displayed a more disorganized actin pattern with many filopodia around the cells. The reduced number of stress fibers, lamellipodia and polarized cells suggest that the cells were not constantly migrating. A similar phenotype has been described for MDA-MB-231 cells treated with strongylophorine-26. The inhibition of cell migration by this marine natural product was in part due to the transient activation of the small GTPase Rho. This protein is important in the regulation of actin dynamics and cell adhesion in migratory cells though the formation of stress fibers and focal adhesions. In addition, Rho, Rac proteins and CDC42 seem to be likely candidates affected by the coatings because of their roles in the induction of lamellipodia and polarization. [45], [46] On the other hand, PLL and PLO had only minor effects on cell morphology, such as a slightly increased cellular area. Interestingly, cells seeded at high confluence on PLL and PLO grew in an organized pattern, where cells were aligned parallel to each other. Filopodia have an important function in the assembling of adherens junctions between cells. Thus, the interdigitation of the abundant number of filopodia observed on the cells grown on PLL and PLO might have contributed to the parallel cell alignment. [43] Hence, the increased CI rate/doubling time relative to control observed by RTCA was probably predominantly caused by a stronger adherence and increased cell surface area attached to the well.

It is well known that physic-chemical characteristics of the substratum can modulate gene expression by remodeling chromatin structure. The reorganization of chromatin may allow access of protein complexes and transcription factors. [47], [48] LNCaP cells grown on PLL displayed elevated DAPI staining intensity compared to the control at 24 h. The increase in DAPI staining might be related to the increase in chromatin condensation. Interestingly, the nuclear area of these cells was larger at 96 h along with reduced DAPI staining. Vergani and collaborators showed that modifications of cell shape directly reflected on the nucleus and the nuclear architecture, followed by chromatin condensation, and finally affecting the transcriptional profile of genes. [49] In addition, the integrins presented by the cells are an effect of the surface substrate which can control the expression levels of their subunits [50].

FN, PLO and PLL were the coating reagents that improved LNCaP cell-substrate adherence. A previous study found that adherence to a FN substrate induced cholesterol (HMGCR activity) and fatty acid synthesis in human fibroblasts and rat hepatoma cells, while a PLL substrate or FN in solution had no effect on these pathways. [26] In addition, FN has been reported to prevent cells from undergoing apoptosis, the mode of death induced by simvastatin. [40] Our studies with the HMGCR inhibitor simvastatin showed that PLL affected the sensitivity to simvastatin. The reason for this somewhat unexpected result is unclear.

The LNCaP cell line is an important model system to study AR-mediated signaling in prostate cancer. It was recently shown that changes to cell-cell contacts and the extracellular matrix altered the response of LNCaP cells to androgens. [28] Importantly, our qRT-PCR analysis revealed that coating with FN, PLL or PLO in general did not alter the response of LNCaP cells to androgens. Although the data shown here investigated only a small cohort of androgen-regulated genes, they strongly suggest that coating with FN, PLL or PLO did not in general change the response of LNCaP cells to androgens, highlighting that these coating reagents are suitable for this important model system of prostate cancer.

## Conclusions

LNCaP cells are the most popular model to study AR-regulated pathways in prostate cancer; however, their use is technically challenging due to their weak cell-substrate adherence. In order to facilitate the use of LNCaP cells in assays that require a strong attachment of the cells to the substrate, five different coating reagents were compared for their impact on various cellular parameters. Coating with PLO, PLL or FN and a cell density of 3.12×10^4^ cells/cm^2^ were found to be ideal with respect to improved adherence and minimal adverse effects on cell behavior.

## Supporting Information

Figure S1 **Cell density effects on cell index (CI) of LNCaP cells seeded on uncoated wells.** Different numbers of cells were seeded on uncoated wells and monitored for 24 h to analyze cell-substrate adherence from the time cells were added to the wells (t = 0) using a real-time cell analyzer (xCELLigence, Roche). Each data point is represented by its means (n = 3) ± SD.
(TIF)

Figure S2 **Wound healing assay by live cell imaging on the IncuCyte system.** Representative images of wounds made on confluent LNCaP cells grown for the indicated times on wells coated as labeled on the left side of the panel. The initial wound contour (t = 0 h) is marked by the dark cell mask and migrating cells are visualized in light gray.
(TIF)

Figure S3 **Sensitivity of LNCaP cells cultured on different coatings to simvastatin.** 24 h after seeding, cells were treated with 98 nM–50 µM simvastatin and growth was monitored for 72 h by RTCA. The IC_50_ was calculated for the indicated time points together with the 95% confidence interval (CI).
(TIF)

Figure S4 **Relative expression levels of AR-regulated genes in response to androgen treatment in the presence of different coatings.** LNCaP cells were grown on uncoated (NC) or coated wells (PLL, PLO or FN) in androgen-depleted medium for 72 h before androgen treatment with R1881 (1 nM) and DHT (10 nM) for 30 h. The expression levels of the indicated genes were analyzed by qRT-PCR, normalized to the housekeeping gene GAPDH and calculated relative to the ethanol uncoated control (NC).
(TIF)

Table S1 **Sequences of the sense and antisense primers used for qRT-PCR experiments.**
(DOCX)

Video S1 **Time-lapse microscopy video of LNCaP cells grown on a well without coating for 96** **h.** Scale bar = 50 µm (20×, Zeiss Axio Observer).
(AVI)

Video S2 **Time-lapse microscopy video of LNCaP cells grown for 96** **h on a well coated with poly-l-lysine.** Scale bar = 50 µm (20×, Zeiss Axio Observer).
(AVI)

Video S3 **Time-lapse microscopy video of LNCaP cells grown for 96** **h on a well coated with poly-l-ornithine.** Scale bar = 50 µm (20×, Zeiss Axio Observer).
(AVI)

Video S4 **Time-lapse microscopy video of LNCaP cells grown for 96** **h on a well coated with fibronectin.** Scale bar = 50 µm (20×, Zeiss Axio Observer).
(AVI)

Video S5 **Time-lapse microscopy video of LNCaP cells grown for 96** **h on a well coated with collagen type IV.** Scale bar = 50 µm (20×, Zeiss Axio Observer).
(WMV)

Video S6 **Time-lapse microscopy video of LNCaP cells grown for 96** **h on a well coated with laminin.** Scale bar = 50 µm (20×, Zeiss Axio Observer).
(AVI)
