# Supplementary figures and images for: Differential Effects of Tissue Culture Coating Substrates on Prostate Cancer Cell Adherence, Morphology and Behavior
Source: PLoS One. 2014 Nov 6;9(11):e112122. doi: 10.1371/journal.pone.0112122 (PMC4223027; doi:10.1371/journal.pone.0112122)

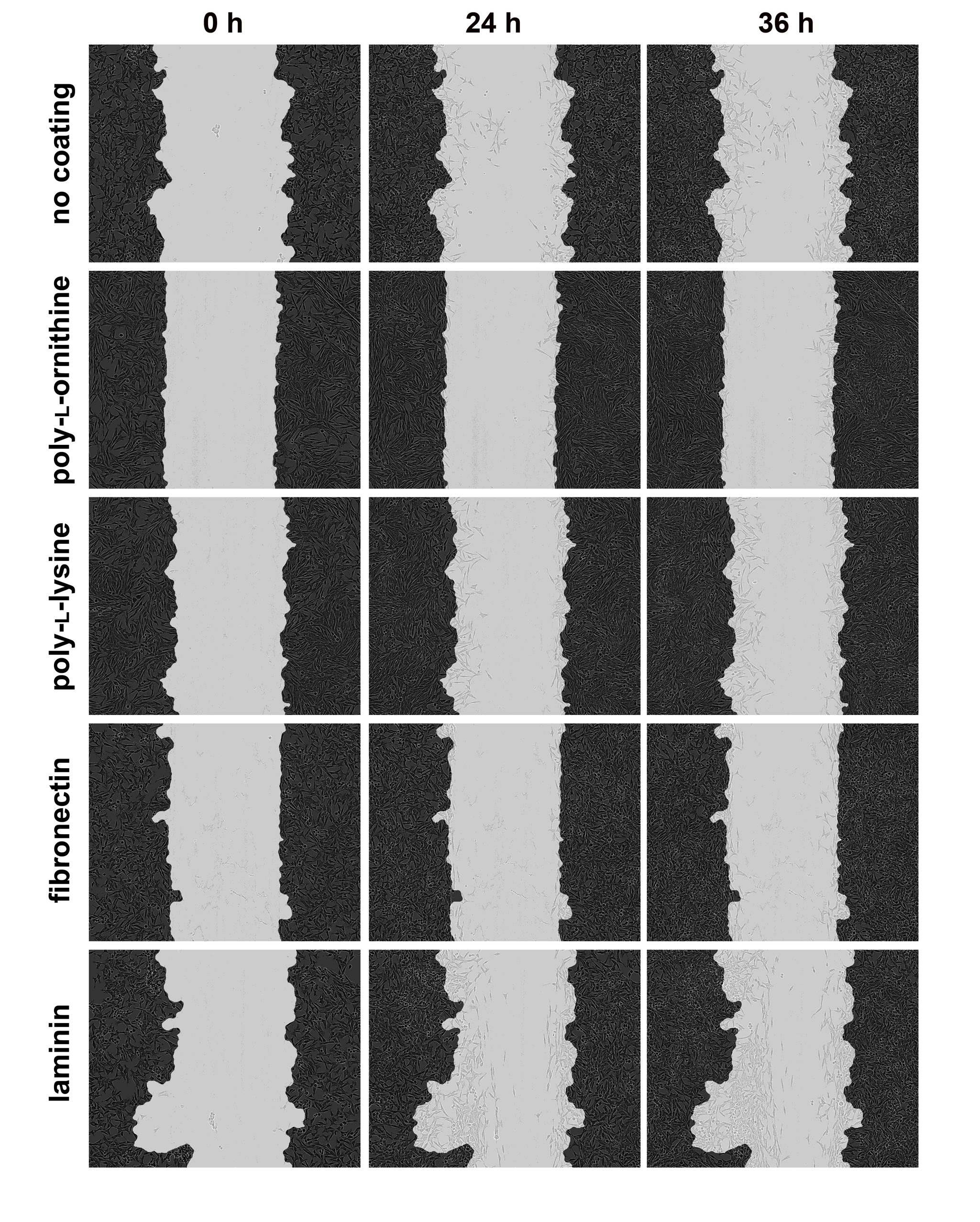

Supplement: Figure S2 — Wound healing assay by live cell imaging on the IncuCyte system. Representative images of wounds made on confluent LNCaP cells grown for the indicated times on wells coated as labeled on the left side of the panel. The initial wound contour (t = 0 h) is marked by the dark cell mask and migrating cells are visualized in light gray. (TIF) [file pone.0112122.s002.tif]

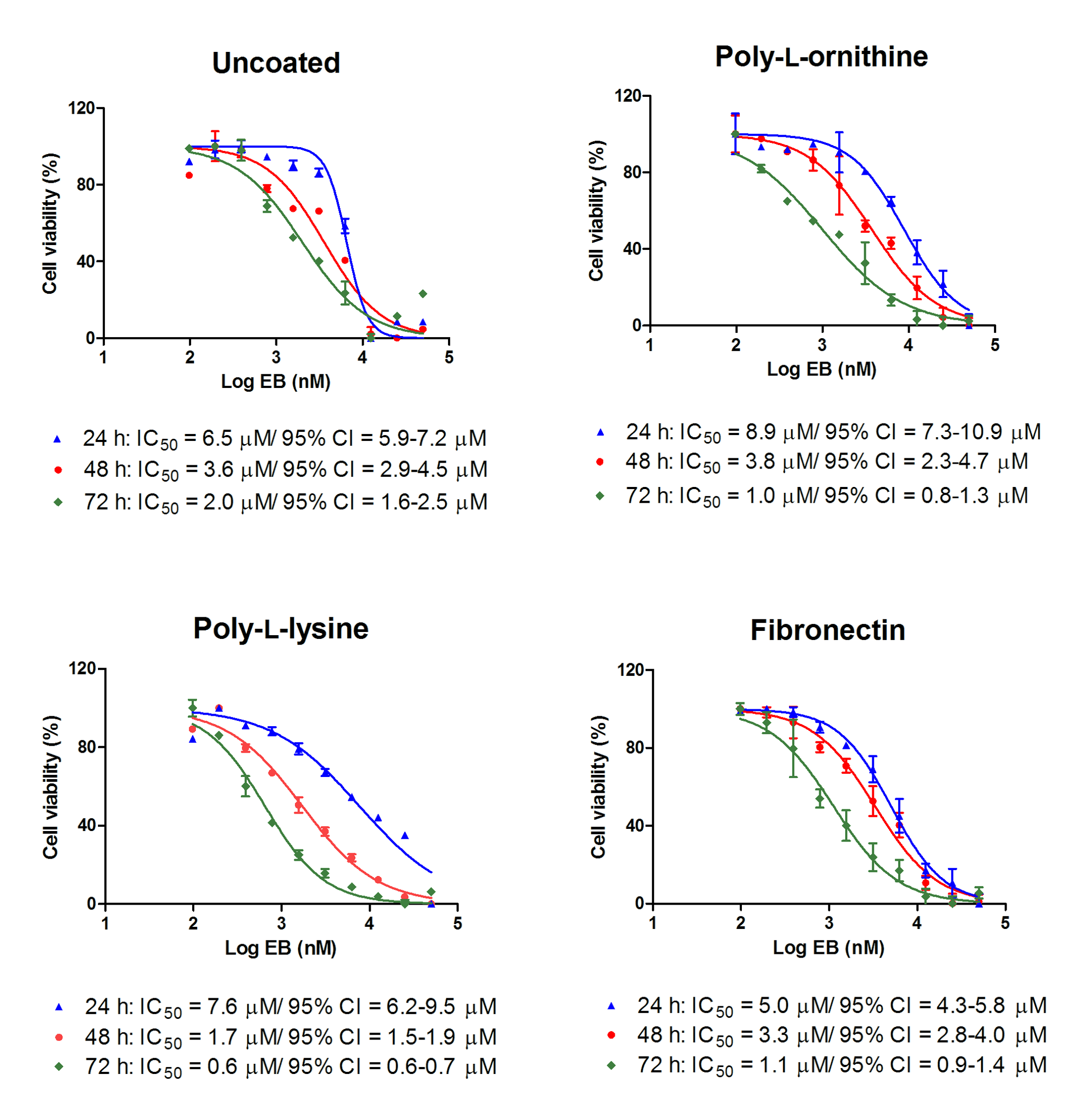

Supplement: Figure S3 — Sensitivity of LNCaP cells cultured on different coatings to simvastatin. 24 h after seeding, cells were treated with 98 nM–50 µM simvastatin and growth was monitored for 72 h by RTCA. The IC50 was calculated for the indicated time points together with the 95% confidence interval (CI). (TIF) [file pone.0112122.s003.tif]

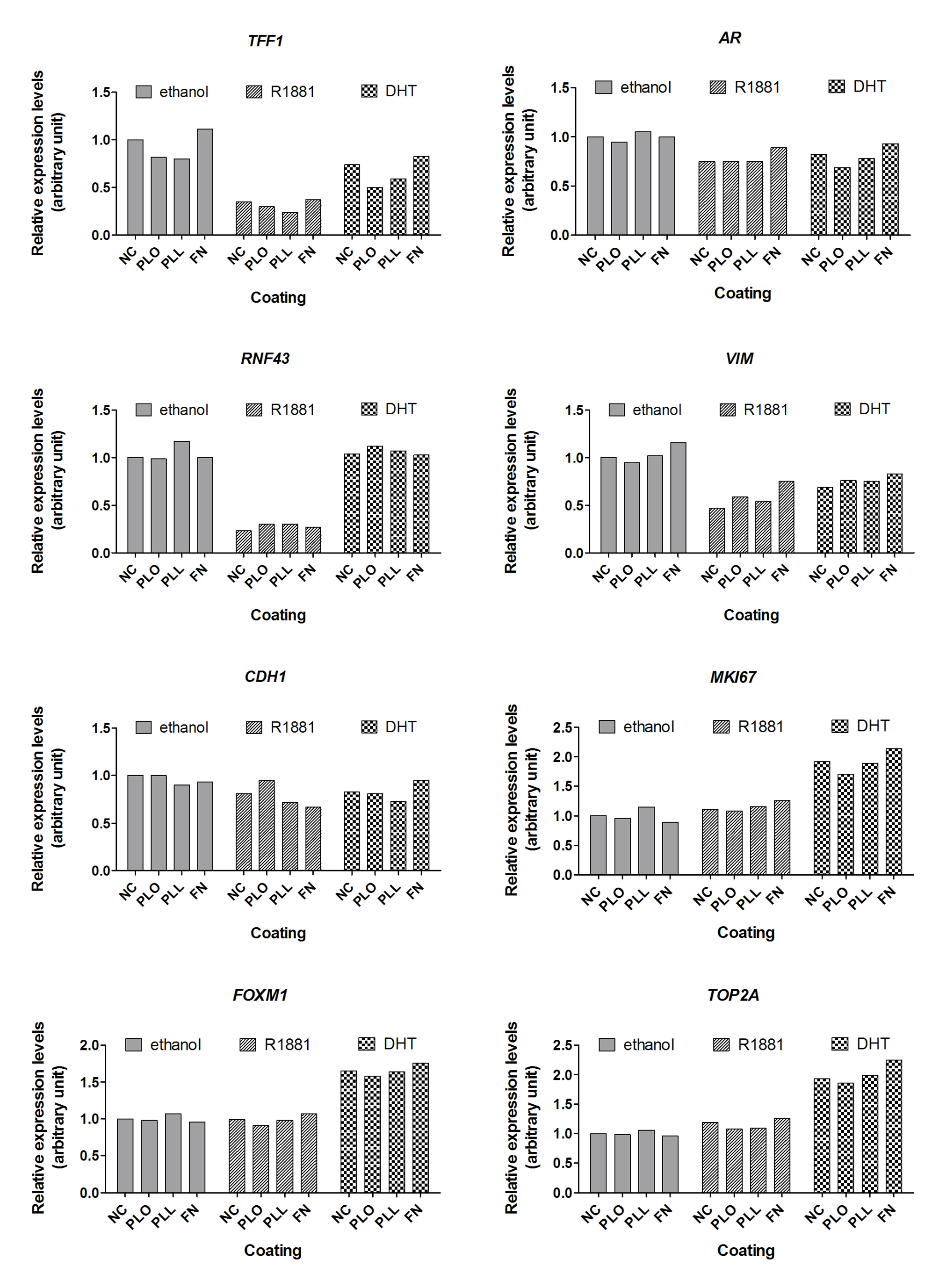

Supplement: Figure S4 — Relative expression levels of AR-regulated genes in response to androgen treatment in the presence of different coatings. LNCaP cells were grown on uncoated (NC) or coated wells (PLL, PLO or FN) in androgen-depleted medium for 72 h before androgen treatment with R1881 (1 nM) and DHT (10 nM) for 30 h. The expression levels of the indicated genes were analyzed by qRT-PCR, normalized to the housekeeping gene GAPDH and calculated relative to the ethanol uncoated control (NC). (TIF) [file pone.0112122.s004.tif]
