# Supplementary material for: Differential Effects of Tissue Culture Coating Substrates on Prostate Cancer Cell Adherence, Morphology and Behavior
Source: PLoS One. 2014 Nov 6;9(11):e112122. doi: 10.1371/journal.pone.0112122 (PMC4223027; doi:10.1371/journal.pone.0112122)
Supplement: Table S1 — Sequences of the sense and antisense primers used for qRT-PCR experiments. (DOCX) [file pone.0112122.s005.docx]

Table S1. Sequences of the sense and antisense primers used for qRT-PCR experiments.

| **Genes** | **Primer pairs** | **Concentration (µM)** |
| --- | --- | --- |
| *RPL32n* | 5’-CCCCTTGTGAAGCCCAAGA  5’-GACTGGTGCCGGATGAACTT | 0.2 |
| *PSA* | 5’-AGTGCGAGAAGCATTCCCAAC  5’-CCAGCAAGATCACGCTTTTGTT | 0.2 |
| *FKBP5* | 5’-AAAAGGCCACCTAGCTTTTTGC  5’-CCCCCTGGTGAACCATAATACA | 0.2 |
| *TMPRSS2* | 5’-CCATTTGCAGGATCCGTCTG  5’-GGATGTGTCTTGGGGAGCAA | 0.2 |
